# Association of rheumatoid arthritis with major adverse cardiovascular events despite normal myocardial perfusion imaging

**DOI:** 10.1016/j.ajpc.2026.101624

**Published:** 2026-04-10

**Authors:** Bailey A. Frohlich, Jacqueline M. Pires, Ioannis Kyrakoulis, Catherine X. Wright, Ibolya Csecs, Ahmed I. Ahmed, Xaviar Jones, Menachem M. Jacobs, Monique Hinchcliff, Edward J. Miller, Attila Feher

**Affiliations:** a Department of Internal Medicine, Yale University School of Medicine, 333 Cedar Street, New Haven, CT 06410, United States; b Section of Cardiovascular Medicine, Department of Internal Medicine, Yale University School of Medicine, 333 Cedar Street, New Haven, CT 06410, United States; c Faculty of Medicine, School of Health Sciences, University of Thessaly, Larissa, Greece; d Section of Rheumatology, Allergy & Immunology, Department of Internal Medicine Yale University School of Medicine, 333 Cedar Street, New Haven, CT 06410, United States; e Department of Radiology and Biomedical Imaging, Yale University School of Medicine, 333 Cedar Street, New Haven, CT 06410, United States

**Keywords:** Rheumatoid arthritis, Myocardial perfusion imaging, Ischemia, Cardiovascular risk, Autoimmune, Coronary artery calcification, Positron emission tomography

## Abstract

**Background:**

Rheumatoid arthritis (RA) is recognized as a cardiovascular risk-enhancing condition. Whether a normal myocardial perfusion imaging (MPI) study confers similar prognostic reassurance in RA as in non-RA patients remains unclear.

**Methods:**

We retrospectively analyzed 282 patients with RA and 282 matched controls without autoimmune disease who underwent SPECT or PET MPI. Matching included age, sex, cardiovascular comorbidities, and stress modality. The primary outcome (MACE) was a composite of cardiovascular-specific mortality, myocardial infarction, late revascularization, or heart failure hospitalization. Multivariable Cox regression and interaction testing between RA and ischemia were performed.

**Results:**

Over a median follow-up of 4.0 years, 95 MACE occurred. Rates of reversible perfusion defects were similar between groups, although coronary artery calcification (CAC) was more prevalent in RA than controls (67% vs. 56%, *p* = 0.048). Despite comparable ischemia burden, RA was independently associated with increased MACE (adjusted HR 1.91, 95%CI 1.24–2.95, *p* = 0.003). RA patients without ischemia had worse outcomes than controls without ischemia (HR 2.16, 95%CI 1.27–3.68, *p* = 0.005), whereas outcomes were similar among patients with ischemia irrespective of RA status. Among patients with detectable CAC, RA was associated with higher risk (HR 1.97, 95%CI 1.07–3.62; *p* = 0.029), and the association between RA and MACE remained significant after additional adjustment for CAC.

**Conclusions:**

In patients referred for stress MPI, RA confers excess cardiovascular risk independent of inducible ischemia and CAC. A normal MPI does not identify a low-risk RA subgroup, supporting aggressive preventive strategies even when perfusion imaging is normal.

**Figure:**
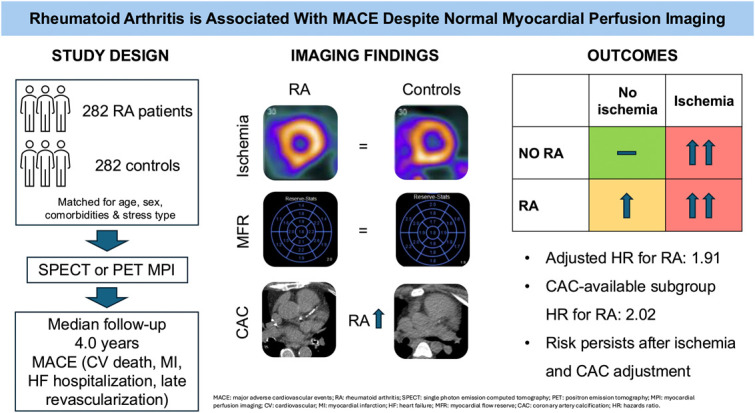
Central illustration.

## 1 Introduction

Rheumatoid arthritis (RA) is associated with approximately a 1.5-fold increase in cardiovascular mortality independent of traditional risk factors [1,2]. Chronic systemic inflammation contributes to accelerated atherosclerosis, endothelial dysfunction, and altered myocardial structure and function [[3], [4], [5], [6]]. As a result, the American College of Cardiology/American Heart Association (ACC/AHA) and the European Alliance of Associations for Rheumatology (EULAR) identify RA as a risk-enhancing factor in cardiovascular risk stratification [7,8].

While myocardial perfusion imaging (MPI) is widely used to risk stratify patients with suspected coronary artery disease (CAD), it remains unclear whether a normal MPI study confers similar prognostic reassurance in patients with RA. Prior studies have reported increased perfusion abnormalities in RA [9,10]. However, whether RA-associated cardiovascular risk is mediated primarily through inducible ischemia, or whether the excess risk persists despite normal perfusion has not been fully characterized.

We therefore examined outcomes in RA patients undergoing stress MPI compared with matched non-RA controls. We specifically tested whether the prognostic implications of ischemia differ by RA status and whether normal MPI identifies a low-risk RA subgroup.

## 2 Methods

### 2.1 Study design and population

We conducted a retrospective, single-center study at Yale New Haven Hospital (New Haven, CT) that was approved by the Yale Institutional Review Board (HIC # 2000021621). The data used in this research are available from the corresponding author upon reasonable request. 282 patients with RA, diagnosed by a rheumatologist, who underwent rest and stress MPI with either ^99m^Tc-tetrofosmin single photon emission computed tomography (SPECT) or ^82^Rubidium (82-Rb) positron emission tomography (PET) between August 2016 and May 2021 were included. Electronic records were manually reviewed to confirm RA diagnosis and medication history.

Additionally, a matched cohort of 282 patients without a history of autoimmune rheumatologic disease (ARD) who had undergone nuclear MPI was matched to RA patients in a 1:1 ratio. Matching was performed using 1:1 nearest-neighbor propensity score matching without replacement in the R statistical environment (R version 3.4; RStudio version 1.1.453; MatchIt package version 3.0.4). Electronic health records of controls were manually reviewed to adjudicate the absence of ARD diagnosis. Propensity scores were estimated using logistic regression with RA as the dependent variable and the following covariates: nuclear perfusion modality (SPECT vs. PET), sex, primary race, history of smoking, transient ischemic attack or stroke (CVA), myocardial infarction (MI), coronary artery bypass surgery (CABG), and percutaneous coronary intervention (PCI), and clinical diagnosis of hypertension, hyperlipidemia, diabetes, heart failure (HF), and chronic kidney disease. Nearest-neighbor matching was performed using the logit of the propensity score as the distance metric. No caliper was applied. The final matched cohort had complete baseline covariate data for all variables included in propensity score matching.

Post-matching balance diagnostics were performed to evaluate matching quality. Covariate balance was assessed using absolute standardized mean differences (SMD) and variance ratios, with SMD <0.10 and variance ratios approximating 1.0 indicating adequate balance. A Love plot was generated to visualize balance across all matched covariates.

### 2.2 Nuclear myocardial perfusion imaging protocol

Image acquisition of SPECT and 82-Rb PET/CT was performed as previously described [11,12]. SPECT imaging was performed using ^99m^Tc-tetrofosmin with a solid state or conventional camera (GE, Healthcare, Haifa, Israel; cameras: GE 530, GE 570C, GE 850, GE Discovery GE Infinia, GE Myosight/CPC, GE Ventri, Philips CardioMD, Siemens eCam). Patients underwent either symptom-limited exercise treadmill stress testing or pharmacological stress test. Patients were selected for exercise or pharmacological stress using clinical parameters. Pharmacologic stress was induced using peripherally administered IV regadenoson (0.4 mg bolus), adenosine (140 μg/kg/min), or dobutamine (up to 40 μg/kg/min) selected based on comorbidity profile. During SPECT, attenuation correction CT was performed based on the camera capability at the performing site. Dynamic rest-stress 82-Rb PET/CT myocardial perfusion imaging was performed on a whole body 2-dimensional (2D)/3D PET camera equipped with a 16-slice CT scanner (Discovery ST, GE Healthcare, Waukesha, Wisconsin).

### 2.3 Image analysis

Nuclear myocardial perfusion images were analyzed using Invia Corridor 4DM v2017 (Ann Arbor, MI) and graded as normal or abnormal perfusion based on the presence of reversible or fixed perfusion defects. Ischemia was defined as presence of a reversible perfusion defect. In patients who underwent 82-Rb PET, stress and rest myocardial blood flow (MBF) and myocardial flow reserve (MFR) were also reported. Global rest and stress MBF (ml/g/min) were calculated using a validated one-compartment kinetic model as previously described [13]. To account for hemodynamic variabilities, rate pressure product (RPP, heart rate x systolic blood pressure) corrected stress, rest MBF and MFR values were also calculated as previously described [14]. Uncorrected MFR <2.0 was considered abnormal [11]. Verification of quantitative values and visual estimation of the presence of perfusion defects and coronary calcification were performed by board-certified nuclear cardiologists. The presence or absence and the extent of CAC was evaluated based on CT acquired for attenuation correction (CTAC) findings as previously described [15]. The visual CAC score was determined by individually assessing calcification in each coronary artery (left main, left anterior descending, left circumflex, and right coronary) and assigning a score of 0 (absent), 1 (mild), 2 (moderate), or 3 (severe). These scores were then summed to produce a total score ranging from 0 to 12 for each patient. Rest and stress left ventricular ejection fractions were derived from ECG gated images.

### 2.4 Laboratory and clinical variables

In both cohorts, the history of presenting symptoms or the indication for MPI and current cardiovascular medications were recorded. Among RA patients, we collected inflammatory markers such as C-reactive protein (CRP) and erythrocyte sedimentation rate (ESR). Patients with RA were further categorized by use of corticosteroids as well as conventional (including methotrexate, leflunomide, sulfasalazine, hydroxychloroquine, chloroquine, azathioprine and mycophenolate), biologic (including tumor necrosis factor alpha inhibitors, abatacept, rituximab, tocilizumab and anakinra), and targeted synthetic (tofacitinib) disease-modifying antirheumatic drugs (DMARDs) at the time of nuclear MPI.

### 2.5 Outcomes variables

Outcomes were ascertained through manual electronic health record review, with follow-up documented for all participants until occurrence of the outcome event, death, or end of available chart data. There was no missing outcome data.

The primary composite endpoint, referred to as the cardiovascular (CV)-specific composite outcome or major adverse cardiovascular events (MACE), was comprised of cardiovascular mortality, MI, HF hospitalization, or late revascularization (>90 days after index stress test). The two secondary composite endpoints were the all-cause composite outcome (all-cause mortality, MI, HF hospitalization, or late revascularization) and HF hospitalization alone.

### 2.6 Statistical analysis

Continuous variables were evaluated for normality using the Shapiro–Wilk test. As all continuous variables were not-normally distributed, they were presented as median [interquartile range (IQR)] and compared using the Wilcoxon rank-sum test. Categorical variables are presented as counts and percentages and were compared with the Chi-square or Fisher’s exact test, as appropriate. Complete case analysis was used for all analyses.

Event-specific incidence rates per 1000 person-years and incidence rate ratios (IRR) comparing the RA cohort to matched controls were calculated for all individual and composite outcomes. Statistical significance was assessed using Poisson regression models accounting for person-years of follow-up. Time-to-event analyses were performed to evaluate the primary composite endpoint. Unadjusted survival analysis was conducted using the Kaplan-Meier method, and statistical comparisons were drawn using the log-rank test.

To identify the impact of ischemia, CAC, inflammatory markers, and DMARD use on cardiovascular outcomes, univariable and multivariable Cox proportional hazards regression models were developed. Variables with *p* < 0.1 in univariable analysis were considered for inclusion in the multivariable models. To avoid overfitting in secondary analyses, additional variable selection was performed by removing variables with the highest p-values despite meeting initial inclusion criteria. The proportional hazards assumption was assessed for all multivariable Cox models in the primary analysis using Schoenfeld residual–based tests and log-log plots and was satisfied for all models (all *p* > 0.05; data not shown).

Prespecified subgroup and stratified analyses were performed according to ischemia status, CAC, and myocardial flow reserve (MFR). Additional models were constructed to evaluate outcomes in patients without ischemia. Sensitivity analyses were conducted using alternative endpoints, including a cardiovascular-specific composite outcome (cardiovascular death, MI, HF hospitalization, or late revascularization) and HF hospitalization alone.

Analyses were conducted with SAS Studio Version 9.4 (SAS Institute Inc., Cary, NC, USA) for the majority of statistical analyses, R (version 4.4.2) with RStudio (Version 2026.01.0 + 392) for Kaplan-Meier curves, Python (v3.14) for post-match balancing, and Stata 19 (StataCorp LLC, College Station, TX, USA) for proportional hazards assumptions testing. A two-sided *p* < 0.05 was considered statistically significant.

## 3 Results

### 3.1 Patient characteristics

A total of 564 participants were included in the study, comprising 282 patients with RA and 282 matched controls. Post-matching diagnostics confirmed excellent covariate balance, with all absolute SMDs below 0.10 and variance ratios approximating 1.0 (Supplementary Table 1; Supplementary Figure 1)*.* Baseline demographic and clinical characteristics are summarized in Table 1. RA patients and controls were well matched, with similar proportions of female (75 % RA vs. 73 % controls) and White participants (68 % vs. 67 %), and comparable median age (68 vs. 67 years) and BMI (30 vs. 29 kg/m²). Traditional cardiovascular comorbidities were similarly distributed between groups, with two-thirds of the participants having hypertension and nearly half having hyperlipidemia. Cardiovascular medication use was also comparable between RA patients and controls, including statins, calcium channel blockers, angiotensin-converting enzyme inhibitors or angiotensin receptor blockers, aspirin, and beta blockers. Most RA patients were receiving disease-modifying therapy, including conventional synthetic DMARDs (62 %), glucocorticoids (35 %), biologic (24 %), and targeted synthetic agents (4 %). Nearly half (44 %) were on combination DMARD therapy, while 17 % were not receiving any DMARDs. Inflammatory markers were elevated in RA patients, with a median ESR of 25.0 [IQR 13.5–46.0] and CRP of 5.2 [IQR 1.9–13.7] (Table 1).

**Table 1:** Baseline characteristics of cohort.

**Characteristic**	**RA (*N*=282)**	**Control (*N*=282)**	**P Value**
**Demographics**			
Age, median [IQR]	67.8 [60.5–74.2]	67.3 [58.2–75.9]	0.576
Female, n ( %)	210 (74.5)	207 (73.4)	0.774
BMI, median [IQR]	30.1 [26.0–35.1]	29.3 [25.8–34.9]	0.619
Primary race, n ( %)			0.934
White/Caucasian	193 (68.4)	190 (67.4)	-
Black	54 (19.2)	52 (18.4)	-
Other	22 (7.8)	26 (9.2)	-
Unavailable	13 (4.6)	14 (5.0)	-
**Cardiovascular Comorbidities, n (%)**			
Hypertension	189 (67.0)	185 (65.6)	0.722
Dyslipidemia	140 (49.7)	126 (44.7)	0.238
Diabetes	52 (18.4)	54 (19.2)	0.829
Smoking history	30 (10.6)	25 (8.9)	0.478
Heart failure	17 (6.0)	13 (4.6)	0.453
Chronic kidney disease	14 (5.0)	12 (4.3)	0.688
Prior MI	18 (6.4)	18 (6.4)	1.000
Prior CABG	13 (4.6)	11 (3.9)	0.677
Prior PCI	19 (6.7)	18 (6.4)	0.865
Prior CVA	11 (3.9)	9 (3.2)	0.649
**Cardiovascular medications, n (%)**			
Statin	137 (48.6)	147 (52.1)	0.400
Aspirin	108 (38.3)	128 (45.4)	0.088
Calcium channel blocker	77 (27.3)	74 (26.2)	0.775
ACE inhibitor	44 (15.6)	50 (17.7)	0.498
ARB	59 (20.9)	52 (18.4)	0.459
Beta blocker	116 (41.1)	109 (38.7)	0.547
**Inflammatory Markers, median [IQR]**			
ESR (*N* = 256)	25.0 [13.5–46.0]		N/A
CRP (*N* = 252)	5.2 [1.9–13.7]		N/A
**DMARD Use, n (%) (*N*=278)**			
Number of DMARDs Used			
0	46 (16.6)		
1	110 (39.6)		
2+	122 (43.9)		
Use of 1+ agent within DMARD class			
Glucocorticoids	98 (35.3)		
csDMARD	171 (61.5)		
bDMARD	67 (24.1)		
tsDMARD (Tofacitinib)	12 (4.3)		
Conventional Synthetic (csDMARDs)			
Methotrexate	82 (29.5)		
Leflunomide	23 (8.3)		
Sulfasalazine	10 (3.6)		
Hydroxychloroquine	81 (29.1)		
Chloroquine	1 (0.4)		
Azathioprine	7 (2.5)		
Mycophenolate	2 (0.7)		
Biological (bDMARDs)			
TNF inhibitors			
Adalimumab	17 (6.1)		
Certolizumab	1 (0.4)		
Etanercept	20 (7.2)		
Golimumab	2 (0.7)		
Infliximab	5 (1.8)		
Non-TNF biologics			
Abatacept	10 (3.6)		
Rituximab	11 (4.0)		
Tocilizumab	2 (0.7)		
Anakinra	1 (0.4)		

Table 1: Categorical variables are summarized as frequency (percentage), and continuous variables are summarized as median (interquartile range) given nonparametric distributions. Group comparisons were conducted using Pearson’s chi square test or Fisher’s exact test for categorical variables when appropriate, and unpaired Mann–Whitney U (Wilcoxon rank-sum) test for continuous variables. For race, “Other” includes Native Hawaiian, Other Pacific Islander, American Indian and Alaska Native. *N* = 282 for each cohort, unless otherwise specified. Abbreviations: RA: rheumatoid arthritis, BMI: body mass index, MI: myocardial infarction, CABG: coronary artery bypass graft surgery, PCI: percutaneous coronary intervention, CVA: cerebrovascular accident, ACE: angiotensin converting enzyme, ARB: angiotensin receptor blocker, ESR: erythrocyte sedimentation rate, CRP: c-reactive protein, DMARD: disease-modifying antirheumatic drug, csDMARD: conventional synthetic DMARD, bDMARD: biological DMARD, tsDMARD: targeted synthetic DMARD, TNF: tumor necrosis factor.

### 3.2 Imaging characteristics

Nuclear myocardial imaging results are summarized in Table 2. Overall, the study included 112 (20 %) PET and 452 (80 %) SPECT studies, and 46 % of the SPECT studies were performed with attenuation correction, evenly distributed between the RA and control groups. Pharmacologic stress was used in the majority of studies (75 %), most commonly with regadenoson. Chest pain was the most frequent indication for imaging (48 %), followed by dyspnea (18 %) and abnormal ECG findings (14 %), with similar indication distributions between groups. Rest and stress hemodynamics, stress left ventricular ejection fraction, and stress ECG responses were comparable between RA patients and controls. There was no significant difference in the prevalence of reversible perfusion defects (ischemia) between RA patients and controls (21 % vs. 17 %, respectively). In contrast, RA patients had a higher prevalence of detectable coronary calcification (67 % vs. 56 %, *p* = 0.048) and a higher median visual coronary calcium score (2 [IQR 0–5] vs. 1 [0–4], *p* = 0.013), although absolute calcium burden was relatively low in both cohorts.

**Table 2:** Myocardial perfusion imaging characteristics.

**Characteristics**	**RA (*N*=282)**	**Control (*N*=282)**	**P value**
**Indication, n (%)**			
Abnormal ECG	38 (13.5)	39 (13.8)	0.902
Dyspnea	53 (18.8)	48 (17.0)	0.583
Heart failure	11 (3.9)	10 (3.6)	0.824
Chest pain	134 (47.5)	138 (48.9)	0.736
Preop evaluation	31 (11.0)	22 (7.8)	0.194
Other	30 (10.6)	38 (13.5)	0.301
**Imaging Modality, n (%)**			0.980
SPECT AC	102 (45.1)	104 (46.0)	
SPECT non-AC	124 (54.9)	122 (54.0)	
PET	56 (19.9)	56 (19.9)	
**Stressor, n (%)**			0.172
Regadenoson	210 (74.5)	201 (71.3)	
Dobutamine/Adenosine	9 (3.2)	4 (1.4)	
Exercise Treadmill	63 (22.3)	77 (27.3)	
**Hemodynamics, median [IQR]**			
Rest SBP, mmHg	134.0 [122.0–152.0]	135 [120.0–150.0]	0.403
Rest HR, bpm	71.0 [64.0–78.0]	72 [63.0–81.0]	0.516
Stress SBP, mmHg	143.5 [126.0–164.0]	146 [129.0–168.0]	0.383
Stress HR, bpm	99.0 [88.0–123.0]	105.0 [90.0–131.0]	0.062
**General Study Results**			
Stress ECG response, n ( %)			0.325
Ischemic	19 (6.7)	25 (8.9)	
Normal	221 (78.4)	193 (68.4)	
Nondiagnostic	34 (12.1)	48 (17.0)	
Equivocal	8 (2.8)	16 (5.7)	
Abnormal perfusion, n ( %)	88 (31.2)	76 (27.0)	0.266
Ischemia, n ( %)	58 (20.6)	47 (16.7)	0.234
**Stress LVEF (RA: *N*=279, Control: *N*=280)**			
Stress LVEF, median [IQR]	68.0 [60.0–74.0]	67.00 [60.0–75.0]	0.998
**Coronary calcification (RA: *N*=158, Control: *N*=160)**			
Detectable coronary calcification, n ( %)	105 (66.5)	89 (55.6)	**0.048**
Visual calcium score, median [IQR]	2.0 [0.0–5.0]	1.0 [0.0–4.0]	**0.013**
**Quantitative PET Results (RA: *N*=53, Control: *N*=56)**			
MBF stress, median [IQR]	2.27 [1.89–2.84]	2.59 [1.83–3.05]	0.156
MBF rest, median [IQR]	1.01 [0.87–1.21]	1.08 [0.80–1.41]	0.737
MFR, median [IQR]	2.20 [1.83–2.53]	2.20 [1.84–2.92]	0.427
Abnormal MFR (<2), n ( %)	19 (35.9)	21 (37.5)	0.858
RPP corrected MFR, median [IQR]	1.65 [1.40–1.89]	1.67 [1.37–2.03]	0.608

Table 2: Categorical variables are summarized as frequency (percentage), and continuous variables are summarized as median (interquartile range) given nonparametric distributions. Group comparisons were conducted using Pearson’s chi square test or Fisher’s exact test for categorical variables when appropriate, and unpaired Mann–Whitney U (Wilcoxon rank-sum) test for continuous variables. *N* = 282 for each cohort, unless otherwise specified. Abbreviations: RA: rheumatoid arthritis, SPECT: single photon emission computed tomography, AC: attenuation correction, PET: positron emission tomography, SBP: systolic blood pressure, HR: heart rate, LVEF: left ventricular ejection fraction, MBF: myocardial blood flow, MFR: myocardial flow reserve, RPP: rate pressure product.

Stress and rest myocardial blood flow did not differ significantly between RA patients and controls who underwent PET studies. MFR was also similar for RA patients compared to non-RA patients when assessed as a continuous measure (2.2 [1.8–2.5] vs. 2.2 [1.8–2.9], respectively; *p* = 0.427) and when dichotomized using a threshold of MFR <2 (36 % vs 38 %, respectively; *p* = 0.858). After RPP correction for both rest and stress, MFR remained statistically comparable (1.65 [1.40–1.89] for RA vs. 1.67 [1.37–2.03] for controls; *p* = 0.608). Given the modest size of the PET subgroup (56 RA and 56 matched controls), these analyses are likely underpowered to detect small-to-moderate differences in MFR, and the absence of statistically significant differences should be interpreted cautiously. Accordingly, these findings should be considered exploratory and hypothesis-generating rather than definitive.

### 3.3 Patient outcomes

Median follow-up for MACE was similar among RA patients and controls (4.1 years [IQR 2.7–5.6] for RA vs. 4.0 years [2.8–5.6], *p* = 0.863). During follow-up, 95 MACE occurred, including 19 cardiovascular deaths, 22 myocardial infarctions (MI), 70 heart failure hospitalizations, and 30 late revascularizations (Table 3). Events were not mutually exclusive. All-cause mortality occurred in 76 patients. The distribution of causes of death was similar between groups (RA vs. control: cardiac 24 % vs. 28 %, non-cardiac 43 % vs. 32 %, and unknown: 33 % vs. 40 %, respectively; overall *p* = 0.647).

**Table 3:** Event-specific incidence rates and incident rate ratios per 1000 person-years.

	**RA (*N*=282)**		**Controls (*N*=282)**			
**Outcome**	**Events (n)**	**Rate per 1000 PY (95% CI)**	**Events (n)**	**Rate per 1000 PY (95% CI)**	**IRR (95% CI)**	**P**
Cardiovascular Death	12	9.67 (4.20–15.14)	7	6.04 (1.57–10.51)	1.60 (0.63–4.07)	**<0.001**
All-Cause Death	51	41.10 (29.82–52.38)	25	21.57 (13.11–30.02)	1.91 (1.18–3.08)	**<0.001**
HF Hospitalization	48	41.74 (29.93–53.55)	22	19.38 (11.28–27.48)	2.15 (1.30–3.57)	**<0.001**
Myocardial Infarction	14	11.61 (5.53–17.70)	8	6.98 (2.14–11.82)	1.66 (0.70–3.97)	**<0.001**
Late Revascularization	18	14.95 (8.04–21.86)	12	10.54 (4.58–16.51)	1.42 (0.68–2.94)	**<0.001**
CV Composite	64	57.44 (43.37–71.51)	31	27.86 (18.05–37.67)	2.06 (1.34–3.17)	**<0.001**

Table 3: Event-specific incidence rates per 1000 person-years and incidence rate ratios (IRR) comparing individual events and the main cardiovascular composite outcome between rheumatoid arthritis (RA) and matched controls (*N* = 282 each). CV Composite includes cardiovascular (CV) death, myocardial infarction, heart failure (HF) hospitalization and late revascularization. IRR p-values derived from Poisson regression models. Abbreviations: RA: rheumatoid arthritis, PY: person-years, IRR: incidence rate ratio, HF: heart failure, CV: cardiovascular.

Event-specific incidence rates per 1000 person-years, accounting for differential follow-up time, were consistently higher in the RA cohort compared to matched controls across all individual outcomes and the primary CV-specific composite endpoint (Table 3). All incidence rate ratios were statistically significant (all *p* < 0.001).

### 3.4 Survival analyses stratified by ischemia

**Fig. 1:**
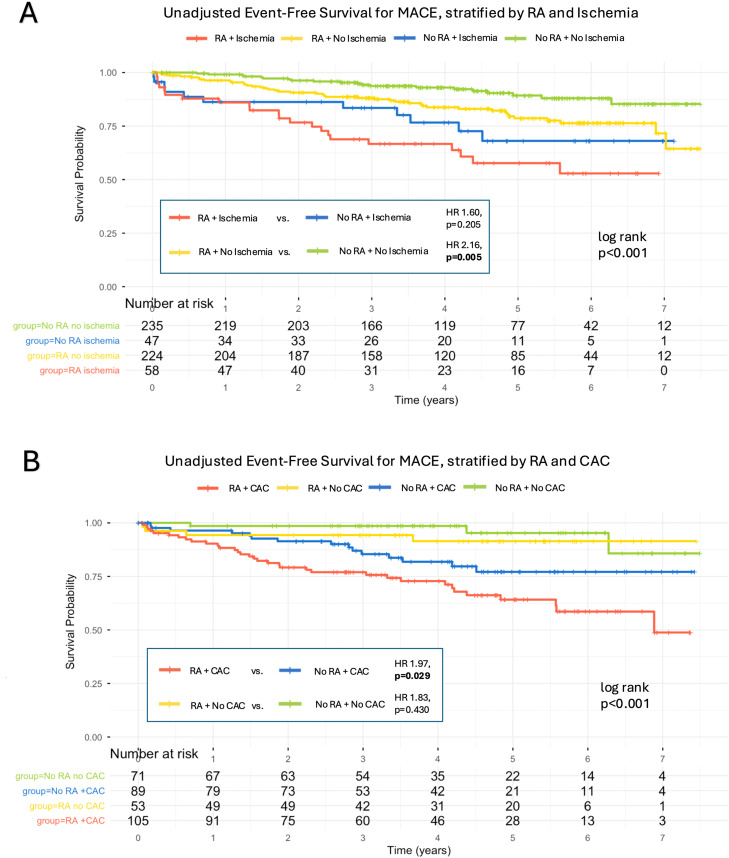
Cardiovascular event-free survival stratified by rheumatoid arthritis status and either ischemia or coronary artery calcification. **Panel A.** Kaplan–Meier curves demonstrating unadjusted event-free survival for the primary CV-specific composite outcome (cardiovascular mortality, MI, HF hospitalization, or late revascularization), stratified by RA and ischemia status. **Panel B.** Kaplan–Meier curves demonstrating unadjusted event-free survival for the primary CV-specific composite outcomes, stratified by RA and CAC status. **Abbreviations:** MACE: major adverse cardiovascular events, RA: rheumatoid arthritis, CV: cardiovascular, MI: myocardial infarction, HF: heart failure, CAC: coronary artery calcification.

Kaplan–Meier survival analyses demonstrated significant differences in event-free survival across groups stratified by RA status and reversible perfusion defects, defined as ischemia (Fig. 1A, log rank *p* < 0.001). RA patients without ischemia experienced worse cardiovascular-specific outcomes compared to controls without ischemia (HR 2.16, 95 % CI 1.27–3.68, *p* = 0.005), whereas survival among RA patients with ischemia was comparable to that of controls with ischemia (HR 1.60, 95 % CI 0.78–3.30, *p* = 0.205).

### 3.5 Multivariable models in the overall cohort

In univariable analyses, RA, ischemia, and multiple cardiovascular comorbidities were associated with MACE (Table 4, Model 1). After multivariable adjustment, both RA and ischemia remained independently associated with adverse events (RA: HR 1.91, 95 % CI 1.24–2.95, *p* = 0.003; ischemia: HR 2.24, 95 % CI 1.45–3.48, *p* < 0.001). No significant interaction between RA and ischemia was observed.

**Table 4:** Cox proportional hazard models for MACE, with and without CAC adjustment.

	Model 1: Entire Cohort, without CAC adjustment (*N* = 564)				Model 2: Subset with available CAC data, with CAC adjustment (*N* = 318)				
	Univariable Regression		Multivariable regression		Univariable Regression		Multivariable regression		
**Factor**	**HR (95% CI)**	**P**	**HR (95% CI)**	**P**	**HR (95% CI)**	**P**	**HR (95% CI)**	**P**	
Age	1.043 (1.023–1.064)	**<0.001**	1.042 (1.019–1.064)	**<0.001**	1.052 (1.026–1.078)	**<0.001**	1.028 (1.000–1.056)		**0.046**
Female	0.971 (0.611–1.543)	0.900			0.940 (0.520–1.702)	0.839			
BMI	1.008 (0.981–1.036)	0.561			1.006 (0.973–1.040)	0.722			
HTN	1.491 (0.944–2.354)	0.087	0.843 (0.513–1.386)	0.501	1.971 (1.041–3.732)	**0.037**			
HLD	0.851 (0.567–1.277)	0.436			0.908 (0.535–1.542)	0.721			
Diabetes	1.772 (1.129–2.783)	**0.013**	1.473 (0.898–2.417)	0.125	2.498 (1.460–4.275)	**<0.001**			
Smoking	1.041 (0.524–2.068)	0.910			0.946 (0.428–2.090)	0.890			
Prior MI	2.036 (1.086–3.818)	**0.027**	1.290 (0.628–2.648)	0.488	2.470 (1.167–5.228)	**0.018**			
Prior CABG	3.705 (2.022–6.788)	**<0.001**	2.326 (1.208–4.481)	**0.012**	5.113 (2.310–11.319)	**<0.001**	2.388 (0.990–5.758)		0.053
Prior PCI	2.673 (1.488–4.801)	**0.001**	1.877 (0.967–3.643)	0.063	2.764 (1.250–6.113)	**0.012**			
Prior HF	4.465 (2.607–7.644)	**<0.001**	4.205 (2.359–7.496)	**<0.001**	4.428 (2.286–8.574)	**<0.001**	3.895 (1.965–7.724)		**<0.001**
Prior CVA	1.624 (0.660–3.996)	0.292			1.611 (0.503–5.161)	0.422			
CKD	1.878 (0.869–4.059)	0.109			2.529 (1.082–5.911)	**0.032**			
CAC	—	—	—-	—	5.121 (2.318–11.310)	**<0.001**	2.738 (1.178–6.363)		**0.019**
RA	2.051 (1.336–3.150)	**0.001**	1.913 (1.240–2.951)	**0.003**	2.190 (1.250–3.838)	**0.006**	2.021 (1.148–3.556)		**0.015**
Ischemia	2.890 (1.893–4.413)	**<0.001**	2.242 (1.445–3.477)	**<0.001**	3.614 (2.110–6.191)	**<0.001**	2.289 (1.271–4.120)		**0.006**
RA*Ischemia				0.706					0.883

Table 4: Multivariable Cox proportional hazards models for the composite endpoint of cardiac-related mortality, MI, HF hospitalization, and late revascularization. Model 1 was performed in the full cohort (*N* = 564; 95 events) without adjustment for coronary artery calcification (CAC). Model 2 was restricted to patients with attenuation-corrected studies (*N* = 318; 56 events), specifically 112 PET and 206 SPECT studies, and included adjustment for CAC. Separate models incorporating an interaction term between rheumatoid arthritis (RA) and ischemia (RA*ischemia) were also evaluated. Abbreviations: CAC: coronary artery calcification; HR: hazard ratio; HF: heart failure; CKD: chronic kidney disease; HTN: hypertension; HLD: dyslipidemia; otherwise as in Table 1.

### 3.6 Multivariable model restricted to patients without ischemia

To further evaluate predictors within the ischemia-negative subgroup, a multivariable Cox proportional hazards model was performed (Supplementary Table 2). Among patients without ischemia, RA remained independently associated with MACE after adjustment for cardiovascular comorbidities (HR 2.03, 95 % CI 1.18–3.48; *p* = 0.010), confirming its incremental prognostic value even in the absence of inducible ischemia.

### 3.7 Sensitivity analyses using alternative endpoints

To assess the robustness of our findings, additional analyses were performed using alternative endpoints (Supplementary Tables 3 and 4). When restricting the composite outcome to all-cause death, MI, HF hospitalization, or late revascularization, RA remained independently associated with increased risk after adjustment for cardiovascular conditions (HR 2.09, 95 % CI 1.44–3.04, *p* < 0.001; Supplementary Table 3, Model 1). Similarly, in analyses limited to HF hospitalization alone, RA demonstrated an independent association with adverse outcomes in multivariable analysis (HR 2.00, 95 % CI 1.19–3.34; *p* = 0.009).

### 3.8 Coronary artery calcification analyses

When stratified by CAC, MACE-free survival differed significantly across groups (Fig. 1B, log-rank *p* < 0.001). Among individuals with detectable coronary calcification, RA was associated with higher risk compared to non-RA patients with CAC (HR 1.97, 95 % CI 1.07–3.62, *p* = 0.029). In contrast, no significant difference was observed in patients without CAC (HR 1.83, 95 % CI 0.41–8.23, *p* = 0.430).

In analyses restricted to patients with available visual CAC evaluation, the association between RA and MACE remained significant even after additional adjustment for CAC (HR 2.02, 95 % CI 1.15–3.56, *p* = 0.015; Table 4, Model 2). Similar results were observed for the all-cause composite outcome (HR 2.26, 95 % CI 1.38–3.71, *p* = 0.001; Supplementary Table 4, Model 2). Among patients without ischemia, RA patients demonstrated modestly higher visual CAC scores compared with controls (Median 2.0 [IQR 0.0–4.0] vs. 1.0 [0.0–3.0], respectively, *p* = 0.038; Supplementary Table 5). In contrast, no differences were observed among patients with ischemia.

### 3.9 PET-derived myocardial flow reserve exploratory analyses

Stratification by RA status and low MFR also demonstrated differences in MACE-free survival (log-rank *p* = 0.015; Supplementary Figure 2). However, outcomes were comparable between RA patients and controls within the same MFR strata, both among those with MFR <2 (HR 1.50, 95 % CI 0.50–4.47, *p* = 0.468) and those with normal MFR (HR 2.99, 95 % CI 0.60–14.80, *p* = 0.181). Among patients without ischemia, reversible perfusion defects, PET-derived MBF, and MFR were similar between RA patients and controls (Supplementary Table 5). Given the limited number of PET studies, these findings should be considered exploratory and hypothesis-generating rather than definitive evidence regarding microvascular function.

### 3.10 RA-specific predictors of events

Within the RA cohort, after adjusting for inflammatory markers and DMARDs, older age and higher ESR remained independently associated with MACE, though the incremental risk per unit was small (age: HR 1.03, 95 % CI 1.00–1.06, *p* = 0.031; ESR: HR 1.01 per unit increase, 95 % CI 1.01–1.02, *p* = 0.003; Table 5).

**Table 5:** Cox proportional hazard models evaluating inflammatory markers and DMARDs among rheumatoid arthritis patients.

	Univariable Regression		Multivariable regression	
Factor	HR (95 % CI)	P value	HR (95 % CI)	P value
Age	1.035 (1.005–1.066)	**0.020**	1.033 (1.003–1.064)	**0.031**
Female	1.029 (0.541–1.959)	0.930		
Body mass index	1.024 (0.991–1.057)	0.153		
ESR	1.015 (1.006–1.023)	**<0.001**	1.013 (1.005–1.022)	**0.003**
CRP	1.002 (0.996–1.008)	0.484		
Steroid	1.367 (0.788–2.374)	0.266		
csDMARD	1.041 (0.597–1.815)	0.888		
bDMARD	0.672 (0.328–1.376)	0.277		
TNF	0.409 (0.147–1.132)	0.085	0.490 (0.175–1.371)	0.174
Non-TNF	1.379 (0.546–3.483)	0.496		
tsDMARD	1.777 (0.429–7.364)	0.428		

Table 5: Multivariable Cox proportional hazards model for the composite endpoint of cardiac-related mortality, myocardial infarction, heart failure hospitalization, and late revascularization among patients with rheumatoid arthritis. The analysis was restricted to patients with available disease-modifying antirheumatic drug (DMARD) and inflammatory marker data (*N* = 237; 53 events). Abbreviations as in Table 1.

## 4 Discussion

In this matched cohort of patients undergoing nuclear stress MPI, RA was associated with a substantially higher risk of MACE despite similar rates of inducible ischemia and comparable PET-derived MFR. Importantly, this finding was observed in a clinically referred population undergoing clinical stress testing, rather than in an unselected RA cohort. The most clinically meaningful observation was that RA patients with normal perfusion did not exhibit the favorable prognosis typically associated with a negative stress perfusion study. In fact, RA patients without ischemia experienced more than twice the risk of adverse events compared with matched non-RA controls without ischemia. Importantly, this association remained robust in multivariable analysis restricted to patients without ischemia, underscoring that RA confers incremental prognostic risk even when inducible ischemia is absent. Thus, within this referred population, normal MPI did not confer the degree of prognostic reassurance traditionally expected. These findings suggest that myocardial perfusion imaging, while useful for detecting flow-limiting coronary disease, does not fully capture the cardiovascular risk conferred by RA.

The excess risk observed in RA was not explained by a higher prevalence of reversible perfusion defects. Rates of ischemia were similar between groups, consistent with prior reports demonstrating that RA-associated cardiovascular risk is not solely mediated by obstructive epicardial disease [9]. There was no significant interaction between RA status and ischemia in multivariable models using a cardiovascular-specific composite endpoint, indicating that RA and ischemia functioned as independent, additive contributors to risk rather than synergistic modifiers. Notably, outcomes among patients with ischemia were comparable regardless of RA status, whereas the divergence in outcomes was most pronounced in the absence of ischemia. Similarly, RA demonstrated an independent association with HF hospitalization alone, suggesting that the excess risk extends beyond atherosclerotic events. Therefore, while ischemia remains an important determinant of adverse events, RA status confers additional, independent cardiovascular risk, emphasizing that traditional perfusion-based risk stratification may underestimate risk in this population.

The mechanisms underlying this residual risk are not fully elucidated and are likely multifactorial. Prior studies have suggested potential contributions from systemic inflammation and non-traditional cardiovascular risk factors [5,[16], [17], [18], [19]]. However, the current study was not designed to directly assess these pathways. While RA patients in our cohort experienced higher rates of heart failure hospitalizations and mortality, similar to prior observations [20,21], our data cannot confirm causality or the specific mechanisms responsible for these outcomes. Observations regarding myocardial remodeling or inflammation-mediated dysfunction should therefore be considered speculative and hypothesis-generating rather than definitive.

Coronary artery calcification was more prevalent in RA, consistent with prior studies demonstrating accelerated subclinical atherosclerosis in inflammatory disease [[22], [23], [24]]. When stratified by CAC, RA was associated with significantly worse outcomes among patients with detectable calcification, whereas outcomes were comparable between RA and non-RA patients in the absence of CAC. Importantly, in multivariable models adjusted for CAC, RA remained independently associated with adverse events. This pattern suggests that RA-related factors, such as systemic inflammation, may contribute to cardiovascular risk beyond the burden of calcified plaque. Although the absolute difference in visual calcium burden was modest, its higher prevalence indicates cumulative atherosclerotic exposure. Nevertheless, the persistence of excess risk after accounting for CAC highlights the potential mediating role of inflammation or other RA-specific mechanisms in driving adverse events.

With respect to coronary microvascular function, PET-derived MFR did not differ significantly between RA and control patients in this cohort. Given the relatively small number of patients undergoing PET imaging, the analysis is underpowered, and these findings should be interpreted as exploratory and hypothesis-generating rather than conclusive. Prior studies have demonstrated impaired microvascular function in autoimmune populations [11,[25], [26], [27]], but the absence of a measurable difference in our study suggests that global reductions in flow reserve may not be the dominant mechanism of excess risk in this referred cohort. Alternatively, RA-associated vascular dysfunction may be regional, inflammatory, or below the resolution of relative perfusion techniques, limiting detection in small subgroups. Therefore, the PET results should be explicitly framed as preliminary and hypothesis-generating, providing a basis for future investigation rather than definitive evidence.

Taken together, these observations challenge the assumption that a normal stress MPI confers uniformly low risk across patient populations. Within a population referred for stress testing, RA appears to represent a condition of persistent residual cardiovascular risk even when inducible ischemia and global flow reserve are preserved. The combination of ischemia-stratified, CAC-adjusted, and cardiovascular-specific analyses supports the conclusion that RA confers independent and clinically meaningful cardiovascular risk beyond what is captured by perfusion imaging alone.

From a preventive cardiology perspective, RA should be considered a risk-enhancing condition for which stress imaging alone is insufficient for comprehensive risk stratification. Importantly, a normal MPI result should not be interpreted as conferring low cardiovascular risk in RA patients, nor should it lead to deferral or de-escalation of preventive therapies when otherwise indicated. Instead, RA status itself should prompt careful attention to guideline-directed risk factor modification, including aggressive management of lipids, blood pressure, and lifestyle factors, irrespective of perfusion findings.

These findings underscore the need to integrate inflammatory disease status into cardiovascular risk assessment frameworks and to recognize that imaging-based reassurance may be incomplete in this population. Future work should clarify whether RA-specific risk algorithms or adjunctive imaging strategies can further refine prevention, but current evidence supports proactive cardiovascular prevention strategies in RA patients regardless of stress imaging results.

### 4.1 Limitations

Our study has several limitations. This was a retrospective, single-center study of patients referred for MPI, introducing potential referral bias and limiting generalizability. Importantly, the study population consisted exclusively of patients referred for stress testing based on clinical suspicion, which introduces significant indication bias. As such, these findings apply specifically to a symptomatic or clinically selected population and should not be extrapolated to unselected RA patients, asymptomatic individuals, or primary prevention settings. Because patients were referred for stress testing based on clinical judgment, the observed event rates likely reflect a higher-risk subgroup of RA patients rather than the broader RA population.

In addition, readers were not formally blinded to clinical characteristics, including rheumatoid arthritis status. Additionally, the PET subgroup was modest in size, therefore mechanistic conclusions regarding microvascular dysfunction should be interpreted cautiously. As standardized metrics of disease duration, disease severity, and seropositivity status were not available, medication use was not randomized and may reflect underlying disease activity, potentially confounding observed associations with cardiovascular outcomes.

RA-specific inflammatory markers (including ESR and CRP) were collected only in the RA group, precluding direct comparison of systemic inflammatory burden between RA patients and controls. Systemic inflammation may also have been present among control patients due to other comorbid conditions, but comparable biomarker data were not available. Consequently, we cannot determine the extent to which differences in inflammatory burden contributed to the observed associations.

While median follow-up was similar between groups, we cannot entirely exclude informative censoring if transfer of care outside our health system was systematically related to health status or RA diagnosis. The lack of longitudinal follow-up data prevented analysis of the temporal relationship between therapy and microvascular function. Although matching helped control for common cardiovascular confounders, unmeasured factors might have influenced the results.

### 4.2 Conclusion

In patients referred for stress MPI, RA is associated with a two-fold increase in MACE independent of inducible ischemia, myocardial flow reserve or coronary calcification. Importantly, a normal myocardial perfusion study does not identify a low-risk RA subgroup. These findings should be interpreted in the context of a clinically selected cohort undergoing stress testing and should not be generalized to asymptomatic or unselected RA populations.

Within referred RA patients, however, the persistence of excess risk despite normal perfusion suggests that clinicians should avoid relying solely on stress imaging results for reassurance. Preventive strategies should be individualized and guided by overall cardiovascular risk assessment rather than deferred solely on the basis of a normal MPI result. Further studies are needed to determine how these findings translate to broader RA populations and to primary prevention settings.

## Declaration of generative AI and AI-assisted technologies

During the preparation of this work, the authors used their institution-specific Clarity Platform to assist with language editing and improving clarity. After using this tool/service, the authors reviewed and edited the content as needed and take full responsibility for the content of the published article.

## Funding

AF received funding from the 10.13039/100000002National Institutes of Health, National Heart, Lung, and Blood Institute (1K23HL168223–01A1). MH received funding from the 10.13039/100000002National Institutes of Health, National Institute of Arthritis, Musculoskeletal and Skin diseases (1R01AR085316–01A1).

## Ethical review

This retrospective, single-center study conducted at Yale New Haven Hospital (New Haven, CT) was approved by the Yale Institutional Review Board (HIC # 2000,025,019).

## CRediT authorship contribution statement

**Bailey A. Frohlich:** Writing – review & editing, Writing – original draft, Visualization, Investigation, Formal analysis. **Jacqueline M. Pires:** Writing – review & editing, Investigation, Data curation, Conceptualization. **Ioannis Kyrakoulis:** Formal analysis. **Catherine X. Wright:** Writing – review & editing, Investigation. **Ibolya Csecs:** Writing – review & editing, Investigation. **Ahmed I. Ahmed:** Writing – review & editing, Investigation. **Xaviar Jones:** Writing – review & editing, Investigation. **Menachem M. Jacobs:** Writing – review & editing, Investigation. **Monique Hinchcliff:** Writing – review & editing, Supervision. **Edward J. Miller:** Writing – review & editing, Supervision. **Attila Feher:** Writing – review & editing, Writing – original draft, Investigation, Formal analysis, Data curation, Conceptualization.

## Declaration of competing interest

The authors declare that they have no known competing financial interests or personal relationships that could have appeared to influence the work reported in this paper.

## Appendix Supplementary materials

Image, application 1

Image, application 2

Image, image 3 dummy alt text

Image, image 4 dummy alt text
