# Supplementary material for: Association of rheumatoid arthritis with major adverse cardiovascular events despite normal myocardial perfusion imaging
Source: Am J Prev Cardiol. 2026 Apr 10;29:101624. doi: 10.1016/j.ajpc.2026.101624 (PMC13329586; doi:10.1016/j.ajpc.2026.101624)
Supplement: Supplementary file 1 [file mmc1.docx]

**Supplementary Figure 1 Title and Legend**

**Title:** Covariate balance in matched cohorts of rheumatoid arthritis patients and controls.

**Legend:** Love plot depicting the absolute standardized mean differences (SMD) for covariates used in propensity score matching between RA patients and matched controls. A vertical line at SMD=0.10 indicates the balance threshold. Imaging modality represents the type of nuclear perfusion study performed (SPECT versus PET).

**Abbreviations:** SMD: standardized mean differences, RA: rheumatoid arthritis, MI: myocardial infarction, CABG: coronary artery bypass graft surgery, PCI: percutaneous coronary intervention, CVA: cerebrovascular accident, CKD: chronic kidney disease, SPECT: single-photon emission computed tomography, PET: positron emission tomography.

**Supplementary Figure 2 Title and Legend**

**Title:** Exploratory analysis of cardiovascular event-free survival stratified by rheumatoid arthritis status and myocardial flow reserve.

**Legend:** Kaplan–Meier curves demonstrating unadjusted event-free survival for the primary CV-specific composite outcome (cardiovascular mortality, MI, HF hospitalization, or late revascularization), stratified by RA and MFR.

**Abbreviations:** MACE: major adverse cardiovascular events, RA: rheumatoid arthritis, CV: cardiovascular, MI: myocardial infarction, HF: heart failure, MFR: myocardial flow reserve.
