# Supplementary material for: Association of rheumatoid arthritis with major adverse cardiovascular events despite normal myocardial perfusion imaging
Source: Am J Prev Cardiol. 2026 Apr 10;29:101624. doi: 10.1016/j.ajpc.2026.101624 (PMC13329586; doi:10.1016/j.ajpc.2026.101624)
Supplement: Supplementary file 2 [file mmc2.docx]

**Supplementary Table 1. Post-Matching Balance Diagnostics for Baseline Covariates.**

| **Covariate** | **Absolute SMD** | **Variance Ratio** |
| --- | --- | --- |
| Imaging Modality | 0.000 | 1.000 |
| Female | 0.024 | 0.974 |
| Primary Race | 0.017 | 0.961 |
| Hypertension | 0.030 | 0.979 |
| Dyslipidemia | 0.099 | 1.011 |
| Diabetes | 0.018 | 0.971 |
| Smoking | 0.060 | 1.177 |
| Prior MI | 0.000 | 1.000 |
| Prior CABG | 0.035 | 1.173 |
| Prior PCI | 0.014 | 1.052 |
| Prior HF | 0.063 | 1.288 |
| Prior CVA | 0.038 | 1.213 |
| CKD | 0.034 | 1.158 |

**Supplementary Table 1:** Post-matching balance diagnostics for baseline covariates between the rheumatoid arthritis and matched control cohorts after 1:1 nearest-neighbor propensity score matching (N=282 matched pairs). Covariate balance was assessed using absolute standardized mean differences (SMD) and variance ratios, with SMD <0.10 and variance ratios approximating 1.0 indicating adequate balance. Imaging modality represents the type of nuclear perfusion study performed (SPECT versus PET). Abbreviations: SMD: standardized mean difference, SPECT: single-photon emission computed tomography, PET: positron emission tomography, MI: myocardial infarction, CABG: coronary artery bypass graft surgery, PCI: percutaneous coronary intervention, HF: heart failure, CVA: cerebrovascular accident, CKD: chronic kidney disease

**Supplementary Table 2. Cox proportional hazard models for MACE in patients without ischemia.**

|  | **Univariable Regression** | | **Multivariable regression** | |
| --- | --- | --- | --- | --- |
| **Factor** | **HR (95% CI)** | **P value** | **HR (95% CI)** | **P value** |
| Age | 1.047 (1.021-1.073) | **<0.001** | 1.045 (1.018-1.073) | **0.001** |
| Female | 1.342 (0.699-2.577) | 0.377 |  |  |
| BMI | 1.011 (0.978-1.044) | 0.524 |  |  |
| Hypertension | 1.790 (1.001-3.201) | 0.050 |  |  |
| Dyslipidemia | 0.883 (0.535-1.459) | 0.628 |  |  |
| Diabetes | 2.125 (1.228-3.679) | **0.007** | 1.838 (1.017-3.322) | **0.044** |
| Smoking | 1.179 (0.508-2.739) | 0.702 |  |  |
| Prior MI | 1.674 (0.671-4.177) | 0.270 |  |  |
| Prior CABG | 3.925 (1.690-9.117) | **0.002** | 2.527 (1.007-6.344) | **0.048** |
| Prior PCI | 3.051 (1.450-6.418) | **0.003** | 2.051 (0.927-4.539) | 0.076 |
| Prior HF | 4.448 (2.259-8.756) | **<0.001** | 3.326 (1.558-7.100) | **0.002** |
| Prior CVA | 1.877 (0.682-5.172) | 0.223 |  |  |
| CKD | 1.721 (0.623-4.750) | 0.295 |  |  |
| RA | 2.163 (1.270-3.684) | **0.005** | 2.026 (1.180-3.479) | **0.010** |

**Supplementary Table 2:** Multivariable Cox proportional hazards model for the composite endpoint of cardiac-related mortality, myocardial infarction, heart failure hospitalization, and late revascularization among patients without ischemia. The analysis was performed in the cohort without ischemia (N=459; 62 events). Abbreviations: RA: rheumatoid arthritis, HR: hazards ratio, BMI: body mass index; otherwise as in Supplementary Table 1.

**Supplementary Table 3. Cox proportional hazard models for all-cause composite outcome, with and without CAC adjustment.**

|  | **Model 1: Entire Cohort, without CAC adjustment**  **(N= 564)** | | | | **Model 2: Subset with available CAC data, with CAC adjustment (N= 318)** | | | | |
| --- | --- | --- | --- | --- | --- | --- | --- | --- | --- |
|  | **Univariable Regression** | | **Multivariable regression** | | **Univariable Regression** | | **Multivariable regression** | | |
| **Factor** | **HR (95% CI)** | **P** | **HR (95% CI)** | **P** | **HR (95% CI)** | **P** | **HR (95% CI)** | **P** | |
| Age | 1.060 (1.042-1.080) | **<0.001** | 1.066 (1.045-1.087) | **<0.001** | 1.067 (1.044-1.091) | **<0.001** | 1.060 (1.034-1.086) | | **<0.001** |
| Female | 0.787 (0.538-1.149) | 0.214 |  |  | 0.837 (0.509-1.376) | 0.484 |  | |  |
| BMI | 1.006 (0.983-1.030) | 0.617 |  |  | 1.002 (0.974-1.032) | 0.869 |  | |  |
| HTN | 1.690 (1.128-2.531) | **0.011** | 0.932 (0.603-1.440) | 0.750 | 2.192 (1.246-3.857) | **0.007** |  | |  |
| HLD | 0.952 (0.673-1.347) | 0.782 |  |  | 0.974 (0.620-1.531) | 0.909 |  | |  |
| Diabetes | 1.904 (1.300-2.789) | **0.001** | 1.744 (1.148-2.652) | **0.009** | 2.545 (1.606-4.034) | **<0.001** | 2.065 (1.278-3.336) | | **0.003** |
| Smoking | 0.928 (0.500-1.722) | 0.813 |  |  | 0.771 (0.370-1.604) | 0.486 |  | |  |
| Prior MI | 1.748 (0.985-3.101) | 0.056 | 1.315 (0.670-2.580) | 0.427 | 2.273 (1.168-4.424) | **0.016** |  | |  |
| Prior CABG | 2.877 (1.621-5.105) | **<0.001** | 1.509 (0.817-2.787) | 0.189 | 3.590 (1.647-7.823) | **0.001** |  | |  |
| Prior PCI | 2.079 (1.193-3.622) | **0.010** | 1.548 (0.812-2.951) | 0.184 | 2.289 (1.099-4.768) | **0.027** |  | |  |
| Prior HF | 3.793 (2.330-6.174) | **<0.001** | 2.828 (1.684-4.750) | **<0.001** | 3.745 (2.059-6.812) | **<0.001** | 2.039 (1.092-3.805) | | **0.025** |
| Prior CVA | 1.951 (0.954-3.991) | 0.067 | 1.791 (0.828-3.876) | 0.139 | 2.454 (1.065-5.655) | **0.035** |  | |  |
| CKD | 2.669 (1.502-4.741) | **0.001** | 2.500 (1.375-4.548) | **0.003** | 3.931 (2.117-7.298) | **<0.001** | 3.270 (1.732-6.172) | | **<0.001** |
| CAC | --- | --- | ---- | --- | 3.958 (2.136-7.335) | **<0.001** | 1.706 (0.880-3.306) | | 0.114 |
| RA | 2.072 (1.433-2.995) | **<0.001** | 2.093 (1.440-3.041) | **<0.001** | 2.398 (1.470-3.912) | **<0.001** | 2.264 (1.380-3.713) | | **0.001** |
| Ischemia | 2.744 (1.901-3.960) | **<0.001** | 2.265 (1.547-3.317) | **<0.001** | 3.079 (1.922-4.933) | **<0.001** | 2.149 (1.310-3.523) | | **0.002** |
| RA*Ischemia |  |  |  | 0.720 |  |  |  | | 0.707 |

**Supplementary Table 3:** Multivariable Cox proportional hazards models for the composite endpoint of all-cause mortality, MI, HF hospitalization, and late revascularization. Model 1 was performed in the full cohort (N=564; 129 events) without adjustment for coronary artery calcification (CAC). Model 2 was restricted to patients with attenuation-corrected studies (N=318; 76 events), specifically 112 PET and 206 SPECT studies, and included adjustment for CAC. Separate models incorporating an interaction term between rheumatoid arthritis (RA) and ischemia (RA*ischemia) were also evaluated. Abbreviations: CAC: coronary artery calcification; HTN: hypertension; HLD: dyslipidemia; HF: heart failure; CKD: chronic kidney disease; HTN: hypertension; HLD: dyslipidemia; otherwise as in Supplementary Table 1.

**Supplementary Table 4. Cox proportional hazards models for heart failure hospitalization.**

|  | **Univariable Regression** | | **Multivariable regression** | |
| --- | --- | --- | --- | --- |
| **Factor** | **HR (95% CI)** | **P value** | **HR (95% CI)** | **P value** |
| Age | 1.059 (1.034-1.085) | **<0.001** | 1.059 (1.032-1.086) | **<0.001** |
| Female | 1.148 (0.657-2.006) | 0.628 |  |  |
| BMI | 1.033 (1.004-1.064) | **0.028** |  |  |
| Hypertension | 2.134 (1.188-3.834) | **<0.001** | 1.131 (0.605-2.116) | 0.699 |
| Dyslipidemia | 0.955 (0.597-1.529) | 0.849 |  |  |
| Diabetes | 2.301 (1.397-3.790) | **0.001** | 1.794 (1.041-3.091) | **0.035** |
| Smoking | 1.130 (0.517-2.469) | 0.759 |  |  |
| Prior MI | 1.847 (0.884-3.859) | 0.103 |  |  |
| Prior CABG | 3.609 (1.792-7.268) | **<0.001** | 2.153 (1.006-4.607) | **0.048** |
| Prior PCI | 1.843 (0.882-3.852) | 0.104 |  |  |
| Prior HF | 6.166 (3.480-10.924) | **<0.001** | 5.004 (2.701-9.268) | **<0.001** |
| Prior CVA | 1.779 (0.648-4.882) | 0.263 |  |  |
| CKD | 2.770 (1.266-6.058) | **0.011** |  |  |
| RA | 2.139 (1.291-3.543) | **0.003** | 1.995 (1.193-3.336) | **0.009** |
| Ischemia | 3.097 (1.904-5.035) | **<0.001** | 2.405 (1.453-3.980) | **<0.001** |

**Supplementary Table 4:** Multivariable Cox proportional hazards model for heart failure hospitalization only. The analysis was performed in the full cohort (N=564; 70 events). Abbreviations are the same as in Supplementary Table 1.

**Supplementary Table 5. Comparison of imaging characteristics by rheumatoid arthritis status, stratified by ischemia.**

|  | **Ischemia** | | | **Non-Ischemia** | | |
| --- | --- | --- | --- | --- | --- | --- |
|  | **RA** | **Non-RA** | **P** | **RA** | **Non-RA** | **P** |
| **CAC Data Available** | **N=33** | **N=26** |  | **N=125** | **N=134** |  |
| Detectable CAC, n (%) | 28 (84.9) | 21 (80.8) | 0.678 | 77 (61.6) | 68 (50.8) | 0.079 |
| Visual coronary calcium score, median [IQR] | 5.0  [2.0-7.0] | 3.5  [1.0-7.0] | 0.424 | 2.0  [0.0-4.0] | 1.0  [0.0-3.0] | **0.038** |
| **PET Studies** | **N=8** | **N=9** |  | **N=45** | **N=47** |  |
| MBF stress, median [IQR] | 1.31  [1.19-1.81] | 1.79  [1.24-2.17] | 0.564 | 2.31  [2.00-2.91] | 2.74  [2.11-3.22] | 0.116 |
| MBF rest, median [IQR] | 0.93  [0.80-1.07] | 1.08  [0.92-1.13] | 0.531 | 1.01  [0.87-1.26] | 1.09  [0.78-1.48] | 0.818 |
| MFR, median [IQR] | 1.53  [1.33-1.99] | 1.84  [1.09-2.92] | 0.810 | 2.33  [1.95-2.58] | 2.26  [1.95-2.92] | 0.662 |
| Abnormal MFR (<2), n (%) | 6 (75.0) | 6 (66.7) | 0.707 | 13 (28.9) | 15 (31.9) | 0.753 |

**Supplementary Table 5:** Imaging characteristics according to rheumatoid arthritis (RA) status, stratified by the presence of ischemia. Coronary artery calcification (CAC) analyses were restricted to studies with attenuation correction (N=318; 112 PET and 206 SPECT). Myocardial blood flow and myocardial flow reserve analyses were limited to PET studies with available data (N=109). Abbreviations: IQR: interquartile range; CAC: coronary artery calcification; MBF: myocardial blood flow; MFR: myocardial flow reserve
