# Supplementary figures and images for: Association of rheumatoid arthritis with major adverse cardiovascular events despite normal myocardial perfusion imaging
Source: Am J Prev Cardiol. 2026 Apr 10;29:101624. doi: 10.1016/j.ajpc.2026.101624 (PMC13329586; doi:10.1016/j.ajpc.2026.101624)

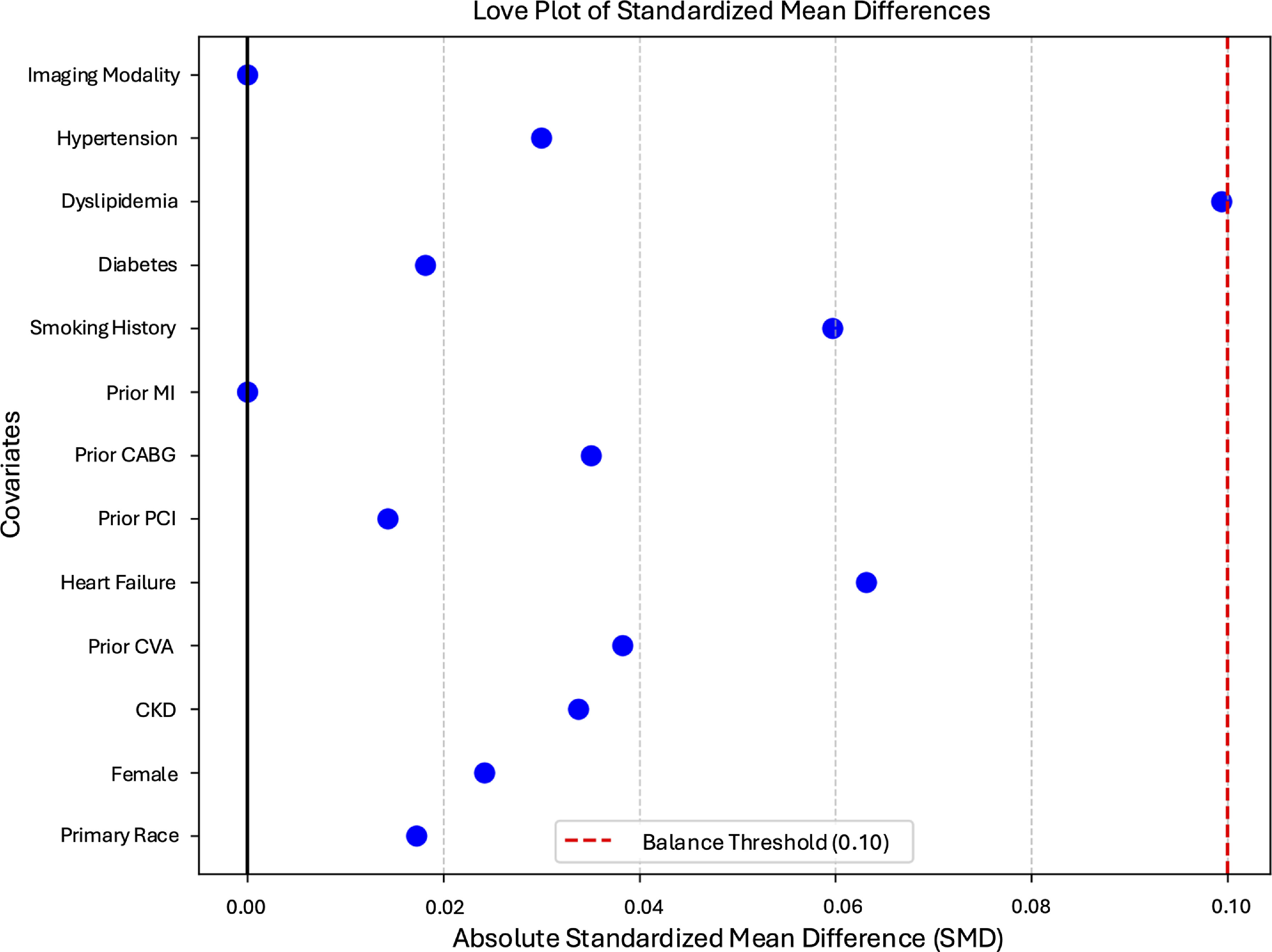

Supplement: Supplementary file 3 [file mmc3.jpg]

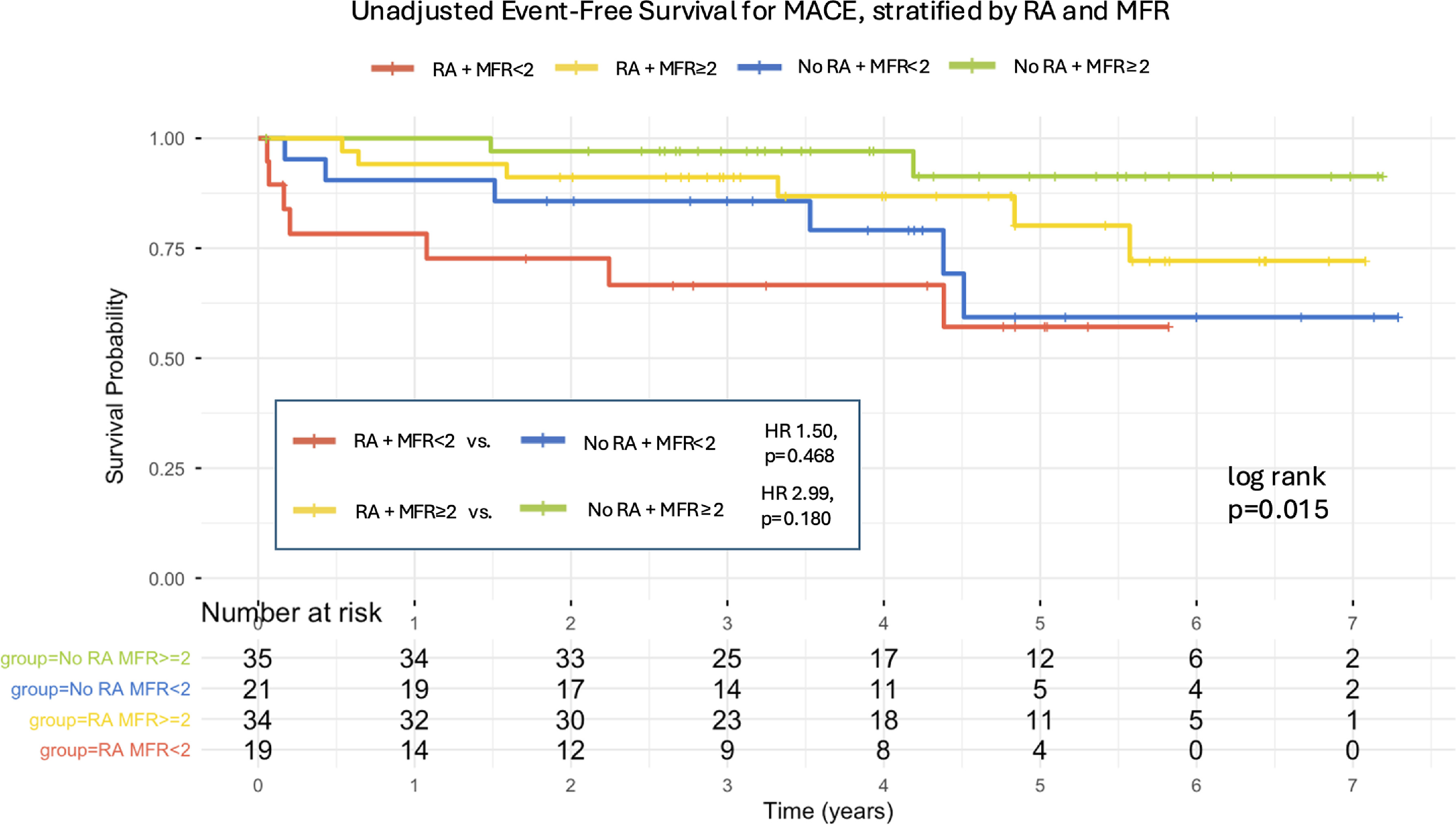

Supplement: Supplementary file 4 [file mmc4.jpg]
